# Willingness to work for sucrose: Impact of schedules, reinforcer alternatives, homeostatic value, and individual differences in male mice

**DOI:** 10.3758/s13420-025-00695-y

**Published:** 2025-11-06

**Authors:** Edgar Arias-Sandoval, Carla Carratalá-Ros, Paula Matas-Navarro, John D. Salamone, Mercè Correa

**Affiliations:** 1 Àrea de Psicobiologia, Universitat Jaume I, Campus de Riu Sec, Castelló, 12071 Spain; 2 Department of Psychological Sciences, Behavioral Neuroscience Program, University of Connecticut, Storrs, CT 06269-1020 USA; 3 Present Address: Department of Psychology, University of Castilla-La Mancha, Albacete, 02071 Spain

**Keywords:** Operant, Mice, Effort, Decision-making, Sucrose consumption

## Abstract

Motivated behavior is characterized by a high degree of behavioral activation and effort. Preclinical models of operant effort-based decision-making are useful for studying motivational symptoms seen in many psychopathologies as well as observing individual vulnerability. In this study, we evaluated the impact of having alternative reward choices with different effort demands on the decision-making process that may lead to individual differences in working. Adult CD1 male mice, trained on a fixed ratio (FR) 8, were divided into two groups: the no-choice (FR8 for 10% sucrose) and choice (FR8 for 10% sucrose plus concurrent free access to 3% sucrose) conditions. After 5 weeks, a progressive ratio (PROG) schedule was introduced under the same choice conditions. Results indicate that only the no-choice group adjusted their work-output after changing from FR8 to PROG. The choice group did not increase lever pressing when PROG was introduced, but it maintained total fluid intake (10% plus 3%). Water restriction increased intake of all fluids in both choice conditions, independently of the effort required. Additionally, a median split based on PROG performance was used to compare high and low performers. Individual differences in lever pressing were seen under both choice conditions, but only high-performers significantly increased work output when PROG was introduced. Among the choice group, high-PROG performers did not increase total volume obtained compared with low-performers. Thus, the choice condition induced individual differences based mostly on willingness to work rather than to obtain the maximum amount of reinforcer. Further studies in both sexes should characterize this “high-worker” profile.

**Supplementary Information:**

The online version contains supplementary material available at 10.3758/s13420-025-00695-y.

## Introduction

Motivated behavior is characterized by a high degree of activity, effort, vigor, and persistence of work output on instrumental tasks (Salamone et al., 2016a, 2016b). The choice to engage in vigorous activities is always undertaken in relation to the possible selection of other more sedentary alternatives. Thus, effort-based decision-making is studied using tasks that offer choices; animals have to choose between high-effort options leading to highly preferred reinforcers versus low-effort options that procure a less preferred reward (Salamone & Correa, 2002). In addition to maze tasks, operant procedures are broadly used for the evaluation of willingness to work for more valued reinforcers. In these studies, rodents have to exert physical effort by lever pressing on a fixed or progressive ratio (FR or PROG) to get access to a highly palatable food or, alternatively, approach and consume the less-preferred laboratory chow (standard food) that is concurrently available during the session (Salamone et al., 1991, 2016a, 2016b). These operant choice tasks are sensitive to motivational manipulations such as food devaluation or food restriction (Nunes et al., 2014; Pardo et al., 2015, 2020; Randall et al., 2014; Yohn et al., 2016).

In previous studies that used the PROG/chow feeding choice task in rats, it was demonstrated that strong and consistent individual differences emerged in terms of the willingness to keep working for the preferred option (i.e., lever pressing for the preferred reinforcer; Randall et al., 2012, 2014). The study of those differences gives us an interesting tool for understanding phenomena such as individual differences in sensitivity to physical effort requirements, which is potentially relevant for modeling symptoms such as anergia, apathy, or fatigue seen in many neurological and psychiatric disorders, and to approach the study of their neural bases and the implementation of potential treatments and preventive strategies (Salamone & Correa, 2024).

Sweet taste also can act as a potent natural reward (Levine et al., 2003). Thus, we also have developed a sucrose version of these operant choice tasks that does not require food restriction (Pardo et al., 2015). In this version, rats lever press for the preferred higher concentration of sucrose or freely consume the lower, less preferred concentration of sucrose during the operant session. In parallel experiments, in which the same sucrose concentrations are given to animals under free access (with no lever pressing requirements), we evaluated the directional aspect of motivation under minimal or no effort demanding conditions. Thus, behavioral variables such as preference between solutions and amount of sucrose consumed can indicate whether animals are oriented toward the reinforcer, and that information can be used to influence the instrumental task in the operant settings (Pardo et al., 2015, 2020; SanMiguel et al., 2018). With this procedure, using the PROG/chow feeding choice task, individual differences also emerged, and the neural substrate that underlies willingness to work for reinforcers that differ in palatability can also be studied (SanMiguel et al., 2018). Pharmacological manipulations that were intended to energize behavior have demonstrated to have a very different pattern of results using both conditions (food in food restricted rats versus liquids with sucrose in non-restricted animals; SanMiguel et al., 2018).

However, most of the research on effort-based decision-making has been done in rats (Cousins & Salamone, 1994; Floresco et al., 2008; Mai et al., 2012; Presby et al., 2021; Salamone et al., 1994). Mice are a very active species that, in experimental settings, show a high level of preference for vigorous activities, such as wheel running or barrier climbing, thus making them well suited to study behavioral activation and effort-based decision-making processes. Studying mice also provides for potential generalizations about these behavioral processes across multiple species. For that reason, some of these effort-based choice paradigms have also been developed for mice and include different types of T-mazes, such as the three-choice T-maze with a running wheel (RW; Carratalá-Ros et al., 2020, 2021a, 2021b; Correa et al., 2016, 2020; López-Cruz et al., 2018; Matas-Navarro et al., 2023) and the T-maze barrier climbing task (Correa et al., 2018; Pardo et al., 2012). These paradigms in mice are sensitive to pharmacological manipulations and to homeostatic and behavioral manipulations such as food restriction or satiation (Carratalá-Ros et al., 2020, 2021a, 2021b; Correa et al., 2016, 2018, 2020; Matas-Navarro et al., 2023; Pardo et al., 2012). All these results are consistent with results in rats observed in both types of T-mazes (Presby et al., 2021; Yohn et al., 2015). However, in mice, the study of individual differences in willingness to exert effort using the choice T-maze paradigms is not feasible since rodents show a very uniform level of response across individuals with very low variability in these experimental settings, which in some cases are divided in discrete trials. A variant of the operant-choice task has been developed for mice (Yang et al., 2020a, 2020b, 2020c).

Thus, the present studies were undertaken to develop and validate a mouse test of effort-related choice behavior using a variant of the operant-choice task developed originally for rats (Pardo et al., 2015; Salamone et al., 1991). We analyzed several conditions in which mice have to exert effort to obtain access to a solution with a high concentration of sucrose. The purpose of this study was to see the impact of having alternative more sedentary choices on the decision-making process, which may influence the appearance of individual differences in working for a reinforcer whose motivational value is mainly based on palatability. Thus, one group of animals was exposed to standard operant procedures, lever pressing for access to the fluid sucrose, with no alternative option to obtain any other reinforcer (no-choice group). The other group was evaluated with a procedure in which the mice have a choice between lever pressing for a high concentration of sucrose or approaching and consuming a lower and less preferred sucrose solution that is freely available concurrently (choice group). Behavioral manipulations that increase fluid motivation were also introduced to assess changes under both choice conditions.

## Materials and methods

### Subjects

CD1 male mice (24–28 g) purchased from Janvier, France S.A. were 4 weeks old upon arrival to the laboratory (*N* = 21). Only males were included in the present work to serve as an initial study to set some of the technical conditions optimal for mice as a species. In subsequent studies the inclusion of both sexes is a paramount practice for the authors. Mice were housed in groups of three per cage and maintained in the colony at 23ºC with 12 h light/dark cycles (lights on at 8:00 h) and standard chow food available ad libitum in the home cage across the entire experiment. Before the operant sessions started, daily average intake of water provided ad libitum for 24 h was measured per animal and was used to establish the total volume of water provided in the home cages (around 5 ml of water per animal per day). This limit in the volume ensured that variability in the home cage water consumption did not affect performance in the operant session. In the water restriction manipulation, we limited the amount of water available in the home cage for the entire group of cage-mates (around 2.5 ml per animal per day) the night before the restriction test was performed. Body weight monitoring was performed on a daily basis to make sure that all animals were always over 85% of the weight of animals the same age. All experimental protocols were approved by the Institutional Animal Care and Use committee of University of Jaume I. All experimental procedures complied with directive 2010/63/EU of the European Parliament and of the Council, and with the “Guidelines for the Care and Use of Mammals in Neuroscience and Behavioral Research” National Research Council 2003, USA. All efforts were made to minimize animal suffering, and to reduce the number of animals used.

### Apparatus and behavioral procedures

#### Operant chambers

Behavioral sessions (once a day, 5 days a week) were performed in operant chambers (28 cm × 23 cm × 23 cm; Med Associates, Fairfax, VT) starting 90 min after the colony lights were on. The operant chambers were equipped with a house-light which remained turned on when the operant session was active. The lever was located on the right side of one of the walls (2 cm above the floor). Completion of the ratio was indicated by a dim yellow light right above the lever, immediately followed by reward availability (access to a retractable sipper of a bottle filled with the high sucrose concentration). In the “choice condition,” the opposite wall of the operant box had another sipper attached to the bottle with the low sucrose concentration. This sipper was freely accessible during the duration of the session. Inputs/outputs of each chamber were controlled by an IBM compatible PC (Med-Associates software). For the selection of sucrose concentration obtained after lever pressing, the duration of sipper availability, and the duration of operant sessions in the experimental phase, see the supplemental material.

#### Ratio training

**Fig. 1:**
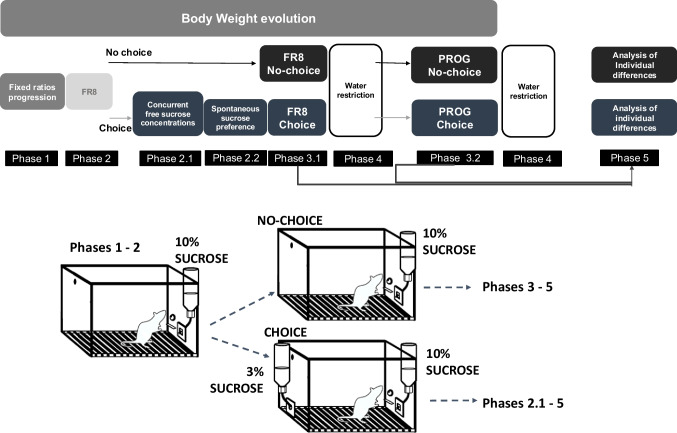
Schematic of the experimental groups and sequence of experiments

For a detailed description of the initial magazine and fixed ratio (FR) 1 training, see the supplemental material. Mice began the FR progression under an FR2 schedule and each new ratio doubled every 4 days (FR2, FR4, and FR8; Exp. 1). After 4 days under FR8 training, mice were split into two groups: A group that continued to have no other way of getting a reward than lever pressing under the FR8 schedule for the 10% solution (FR8/no-choice group), and the other half of the animals were moved into an FR8/sucrose drinking choice condition. The cages containing the animals were randomly assigned to one of the two groups, and a Student’s *t* test for unpaired samples shows that the average baseline (4 days of FR8) was not statistically different across the two groups (no choice: average lever presses 97.83 ± 15.15, and Choice: 87.20 ± 11.04), *t*(20) = 0.558, *p* =.5827. Under the choice condition the 10% sucrose concentration bottle was available after completing each ratio, but concurrently in the chamber there was a bottle containing a lower concentration of sucrose freely available during the entire session. This “FR8/choice group” was the one used for Phases 2.1 and 2.2, in which we assessed which freely available sucrose concentration (1% or 3%) was enough to reduce lever pressing for the high 10% sucrose. After that, animals in the choice group concurrently had free access to the 3% sucrose sipper for the rest of the experiment. Animals spent 5 weeks on FR8/choice or FR8/no-choice conditions, and then a progressive ratio (PROG/choice or PROG/no choice) was required for both groups Phase 3.1. The PROG schedule was based on the same features as the fixed ratio progression, starting with a fixed ratio of 2, being that this ratio doubled every time the animal achieved 3 consecutive reinforcers (3 × FR2, 3 × FR4, 3 × FR8, 3 × FR16, 3 × FR32...). The PROG schedule operated such that the animal needed to maintain responding in order to avoid a time-out; if 2 min occurred without a response, then the light over the lever was turned off and the sippers were retracted permanently. This setting ensured that the session ended when the mice reached the point of ratio strain (unwillingness to keep responding). For the water restriction phases under FR8 and PROG schedules were performed on the last weekend of each schedule. For a detailed progression of the phases of the study, Fig. 1 represents the timeline and the sequence order.

### Statistical analyses

**Fig. 2:**
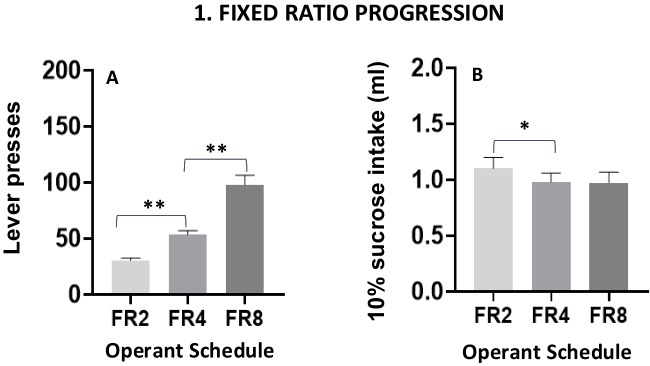
Effect of different operant fixed ratio schedules performed in consecutive weeks on lever presses (**A**) and 10% sucrose intake (**B**). Bars represent the mean ± *SEM* number of lever presses or ml consumed in 15 min (*N* = 23). *p <.05, **p <.01, significant differences between schedules

**Fig. 3:**
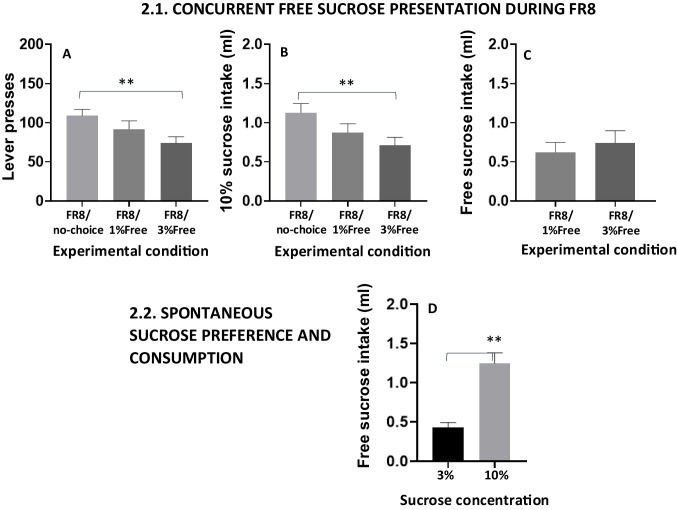
Upper part: Effect of introducing free choice conditions during an operant FR8 session (*N* = 11). Lever presses (**A**), operant-dependent 10% sucrose intake (**B**), and free sucrose intake (**C**). Bars represent the mean ± *SEM* number of lever presses or ml consumed in 15 min. Lower part: (**D**), Effect of concurrent free access presentation in the operant box of two concentrations of sucrose (10% and 3%) on volume consumed. Bars represent the mean ± *SEM* of ml consumed in 15 min. **p <.01, significant differences between experimental conditions

**Fig. 4:**
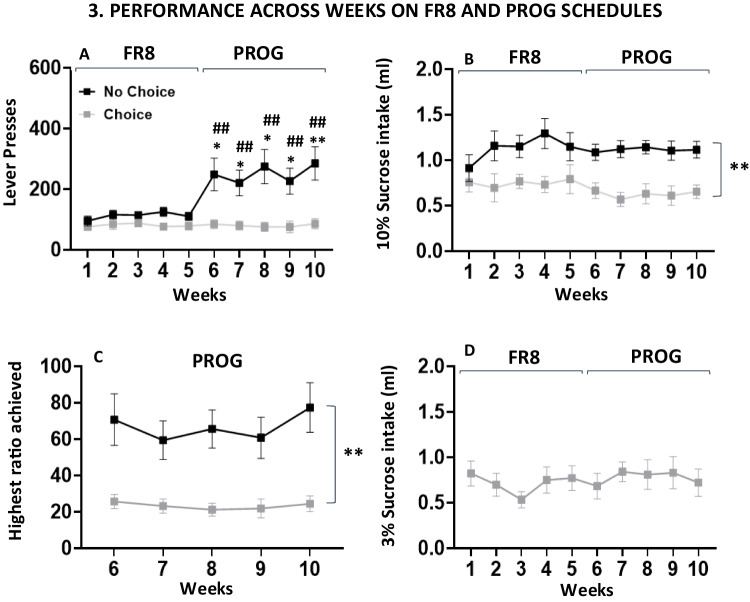
Effect of choice conditions during operant sessions with different work requirements (FR8 and PROG schedules,* N* = 22). Data represent the mean ± *SEM* number of lever presses (**A**), 10% sucrose ml consumed (**B**), highest ratio achieved during the PROG weeks (**C**), and free 3% sucrose ml consumed for the choice group (**D**) in 15-min sessions. *p <.05, **p <.01, significant differences between choice groups. ##*p* <.01, significantly different from last FR8 week

**Fig. 5:**
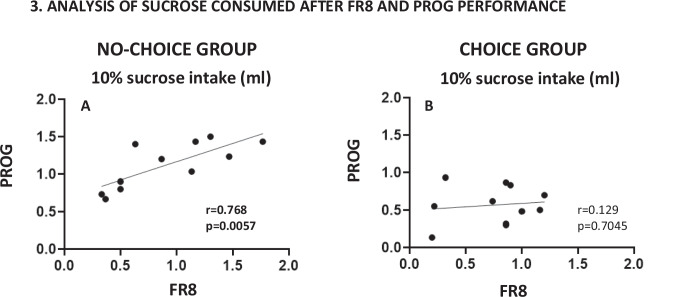
Linear correlation between the average of the 10% sucrose solution consumed by individual mice during the last week of each schedule. Individual data for the no-choice group (**A**, *n* = 11) and the choice group (**B**, *n* = 11)

**Fig. 6:**
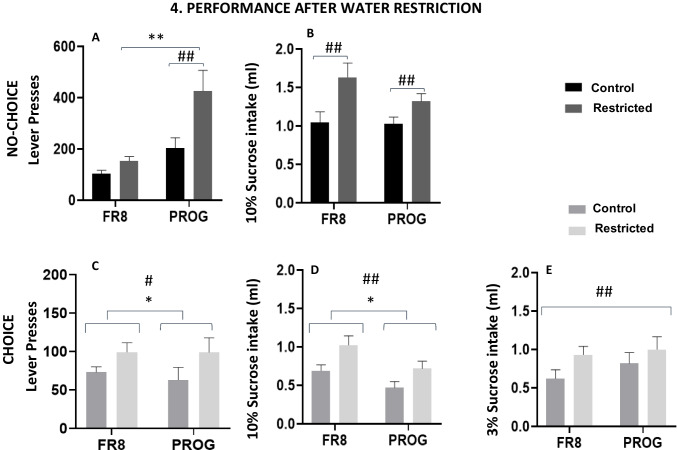
Effect of water restriction (*N* = 22; *n* = 11 for choice condition and *n* = 11 for no choice condition) on FR8 lever presses (**A**, **C**), operant-dependent 10% sucrose intake (**B**, **D**), and free 3% sucrose intake (**E**). Bars represent the mean ± *SEM* number of lever presses or ml consumed in 15 min. *p <.05, **p <.01 significant differences between schedules. #*p* <.05, ##*p* <.01 significant differences between restriction conditions. In **C**–**E**, # denotes significance for the main factor water restriction, and * denotes significance for the main factor operant schedule

**Fig. 7:**
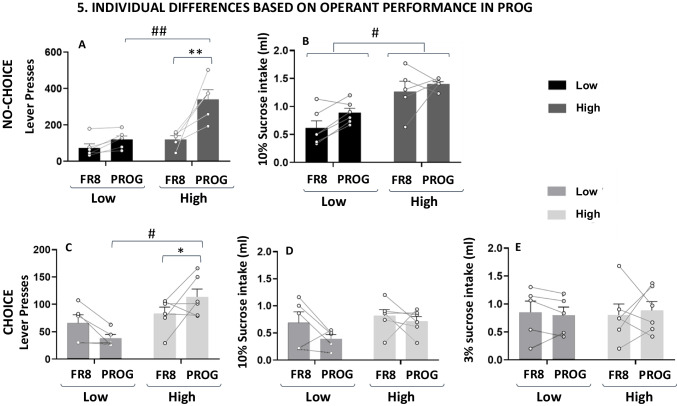
Analyses of individual differences based on lever presses for the last week of PROG/choice (*n* = 11) and PROG/no-choice (*n* = 11) conditions. Bars represent the mean ± *SEM* number of lever presses (**A**, **C**), operant-dependent ml of 10% sucrose consumed (**B**, **D**), and ml of free 3% sucrose consumed (**E**) for a session of 15 min. *p <.05, **p <.01 significant differences between schedules. #*p* <.05, ##*p* <.01 significant differences between performers. In **B**, # denotes significance for the main factor performer

**Fig. 8:**
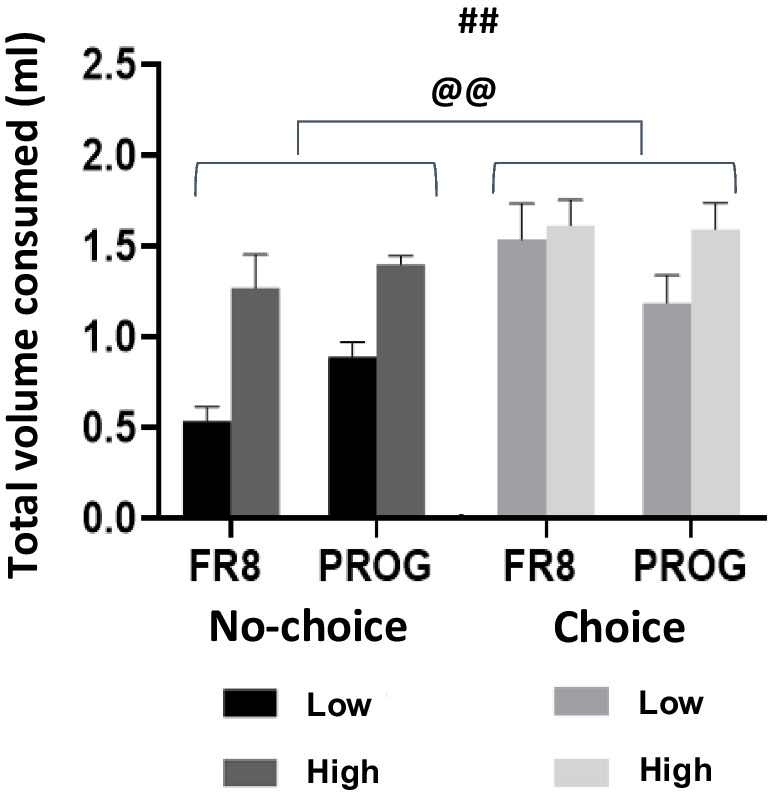
Total volume consumed (*N* = 22) during the operant session comparing high and low performers in both choice groups and both schedules. Data represent mean ± *SEM* of total volume (10% + 3% solutions) consumed during the operant session. ##*p* <.01 significance for the main factor type of performer, @@*p* <.01 significance for the main factor choice condition

**Fig. 9:**
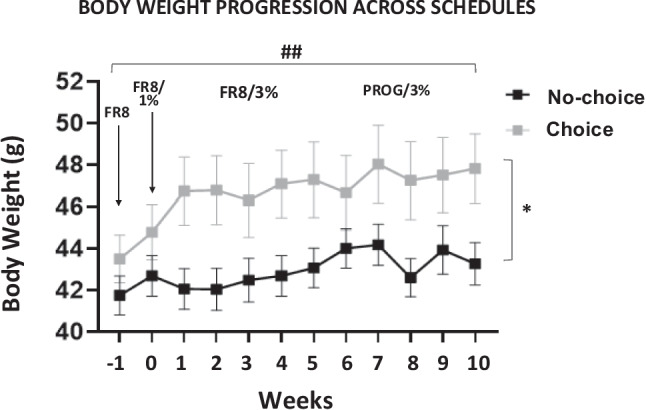
Body weight evolution for the two groups (*N* = 22) across weeks. Data represent mean ± *SEM* of weekly body weight in grams. *p <.05 significant effect of the factor group

For all statistical analyses Prism 8.0 software (GraphPad, San Diego, CA, USA) was used. Repeated-measures analysis of variance (ANOVA) was used in Phases 1 (Figs. 2A–B, main factor: fixed ratio schedules), Phase 2.1 (Figs. 3A–B, main factor: free option concentrations), and Phase 3 (Fig. 4D, main factor week of training). Paired Student’s *t* tests were used in Phases 2.1. (Fig. 3C, comparing free option concentrations), and 2.2 (Fig. 3D, comparing concentrations of sucrose). Two-way factorial ANOVAs were used for Phase 3 (Figs. 4A–C. Choice Group × Week), Phase 4 (Figs. 6A–E. Operant Schedule × Restriction Condition), and Phase 5 (Figs. 7A–E. Operant Schedule × Type of Performer), and also for the body weight data (Fig. 9. Choice Group × Week). Additional correlational analyses were used in Phase 3 (Figs. 5A–B). In Phase 5 (Fig. 8), a three-way factorial ANOVA was used (Schedule × Performer × Choice Condition). As a measure of effect size, partial eta squared (η_p_^2^) was reported. When the overall ANOVA was significant, nonorthogonal planned comparisons using the overall error term were used to compare each experimental condition (Keppel, 1991). Student *t* tests for paired samples were used in Phase 2.1 (Fig. 3C) and 2.2 (Fig. 3D), and Cohen’s *d* was reported as a measure of effect size. For these comparisons, α level was kept at 0.05, and the number of comparisons was restricted to the number of experimental manipulations minus one. All data were expressed as mean (± *SEM*), and significance was set at *p* <.05.

## Results

### Phase 1. Fixed ratio progression: Lever pressing performance and sucrose consumed

Repeated measures ANOVA was performed to compare the impact of incremental fixed ratio changes over several days on work performance and sucrose consumption. Results showed a significant increase in lever presses, *F*(2,44) = 83.25, *p* <.01, η_p_^2^ = 0.791 (Fig. 2A) across schedules. Planned comparisons showed significant differences in lever presses between FR2 and FR4 and between FR4 and FR8 (*p* <.01 in both cases). A repeated-measures ANOVA of the incremental fixed ratio levels for the dependent variable 10% sucrose intake (Fig. 2B) also showed significant differences between schedules, *F*(2,44) = 3.90, *p* <.05, η_p_^2^ = 0.151. In this case, planned comparisons yielded significant differences between FR2 and FR4 (*p* <.05), but not between FR4 and FR8. Thus, in going from the FR4 to the FR8 schedule, animals doubled the work to maintain access to the same volume of sucrose solution.

### Phase 2. Impact of different sucrose alternatives on work performance and consumption

#### Phase 2.1. Effect of concurrent free sucrose presentation on FR8 performance and sucrose consumption

After separating the two choice groups, the FR8/choice group had the 10% sucrose concentration as the high-effort/high-reward option and also a 1% sucrose concentration as the low-effort/low reward option for 1 week (5 days per week). Then, the freely available 1% sucrose concentration was replaced by a concentration of 3% during 1 more week. Lever presses and sucrose consumption from the last week of no-choice condition, the week of 1% concurrent solution and the first week of 3% concurrent solution were compared. The FR8/no-choice group continued to train during these 2 weeks (total of 10 days; data not shown).

The repeated-measures ANOVA yielded a significant effect of the experimental condition, *F*(2,20) = 4.76,* p* <.05, η_p_^2^ = 0.322, showing a decrease in lever presses when a concurrent free choice was available (Fig. 3A). Planned comparisons showed that this difference was significant (*p* <.01) for FR8/Free 3% condition when compared to FR8/no-choice. Regarding 10% sucrose intake, a one-way repeated-measures ANOVA also displayed a significant decrease on intake, *F*(2,20) = 6.85, *p* <.01, η_p_^2^ = 0.407, when a concurrent free choice was available (Fig. 3B). Planned comparisons showed significant differences between no-choice and 1% sucrose choice (*p* <.01), and between 1 and 3% sucrose concentrations (*p* <.05). A paired *t* test of the free sucrose intake data revealed that no differences were found between the free solutions 1% and 3% concentrations, *t*(10) =  − 1.340, *p* =.20, *d* =  − 0.404 (Fig. 3C). Thus, although animals did not show differences in consumption between the two free sucrose solutions, only 3% was able to change willingness to lever press when presented as the free option. Thus, for the rest of the experiments, 3% sucrose was the concentration used as the free option.

#### Phase 2.2. Preference for 10% versus 3% sucrose under free-access conditions

Once the 3% sucrose concentration was chosen as the low-effort/low-reward option for the rest of the experiments, we compared spontaneous preferences for sucrose concentration between 10% versus 3% to make sure that animals had a clear preference for the high concentration so they were willing to work for it. Thus, for 4 days, animals had concurrent access to both concentrations of sucrose (10% and 3%) freely accessible in two bottles placed in opposite walls of the operant chambers. Average baseline for the 4 days of presentation between both sucrose concentrations was analyzed. A paired *t* test showed a significant difference between milliliters consumed of 10% sucrose in comparison with 3% sucrose, *t*(10) = 5.093, *p* <.01, *d* =  − 1.535 (Fig. 3D).

### Phase 3. Increase in work demand: Impact across “choice” and “no-choice” groups

After the FR8 performance, both groups; “no-choice” (*n* = 11) and “choice” (*n* = 10), were exposed to the PROG schedule. For the dependent variable lever presses, a two-way factorial ANOVA with a between groups factor (“no-choice” vs. “choice”) and a within factor (week) showed significant effects of the choice condition, *F*(1,20) = 12.89, *p* <.01, η_p_^2^ = 0.392, of test week, *F*(9,180) = 6.69, *p* <.01, η_p_^2^ = 0.251, and also a significant interaction, *F*(9,180) = 6.66, *p* <.01, η_p_^2^ = 0.250. Planned comparisons revealed that the “no-choice” group increased lever presses when the instrumental response requirement was higher (i.e., change from Week 5 of FR8 to the other weeks of PROG, *p* <.05 for Weeks 6–9 and *p* <.01 for Week 10; Fig. 4A). There were no significant differences across weeks on the same schedule and among the same choice group. Moreover, the “no-choice” group significantly increased lever presses in comparison with the “choice” group when the operant schedule changed from FR8 to PROG (*p* <.01 for all weeks).

For the variable 10% sucrose intake (data shown in Fig. 4B), the two-way factorial ANOVA showed a significant effect for the factor choice, *F*(1,20) = 11.10, *p* <.01, η_p_^2^ = 0.357), but no significant effect of week, *F*(9,180) = 1.25, *p* =.26, η_p_^2^ = 0.059, and no interaction, *F*(9,180) = 1.27, *p* =.2, η_p_^2^ = 0.06. Among the “choice” group, we also analyzed the effect of week on free concurrent 3% sucrose consumption (Fig. 4D). The repeated-measures ANOVA did not show any significant effect of weeks on the intake, *F*(1,9) = 0.94, *p* =.48, η_p_^2^ = 0.087. Thus, in the choice group the sucrose consumed was stable across the weeks of training on FR8 and PROG. Finally, we also analyzed the effect of choice condition on the highest ratio achieved under the PROG schedule. The two-way factorial ANOVA showed a significant effect for the factor choice, *F*(1,20) = 13.49, *p* <.01, η_p_^2^ = 0.403, but no significant effect of the week, *F*(4,80) = 2.47, *p* =.09, η_p_^2^ = 0.011, nor a significant interaction, *F*(4,80) = 1.38, *p* =.24, η_p_^2^ = 0.006 (Fig. 4C). This last result confirms the stability of work output since the first week of PROG.

To further understand the impact of the schedule on sucrose consumption among the different choice groups, we did a correlation between the individual level of 10% sucrose intake when the animal responded on the last week of FR8 and the last week of PROG performance among the “no-choice” and “choice” groups separately. The correlation was significant in the “no-choice” group (*r* =.768, *p* =.005), indicating that when animals have no choice, they adjust their operant behavior to the amount of fluid that they aim to achieve. However, among the “choice” group, the correlation was not significant (*r* =.129, *p* =.704), suggesting that when animals have a concurrent free option, they regulate their operant output in a less predictable way, probably because they have the free source of reinforcer (Figs. 5A–B).

### Phase 4. Effect of water restriction on performance

We evaluated the impact of increasing motivation for fluid in both groups separately by limiting the amount of water available in the home cage (around 2.5 ml per animal per day) the night before the restriction test was performed. This restriction was implemented after the operant session on the 5th day of the last week of FR8 and PROG schedules (see Fig. 1). Thus, Day 5 (operant session before water restriction) was considered as the control for the restriction on Day 6.

In the no-choice group (Fig. 6A–B), the two-way factorial ANOVA (Water Restriction × Operant Schedule) for lever presses yield a significant effect of restriction, *F*(1,10) = 34.30, *p* <.01, η_p_^2^ = 0.774, operant schedule, *F*(1,10) = 11.39, *p* <.01, η_p_^2^ = 0.532, and a significant interaction, *F*(1,10) = 20.48, *p* <.01, η_p_^2^ = 0.672. Planned comparisons revealed that mice lever pressed more when they were water restricted in comparison with baseline conditions under the PROG schedule (*p* <.01). In addition, under water-restriction conditions, mice lever pressed more in the PROG schedule compared with the FR8 schedule (*p* <.01). As for the 10% sucrose intake dependent variable, the two-way factorial ANOVA did not show a significant effect of operant schedule, *F*(1,10) = 2.681, *p* = 0.133, η_p_^2^ = 0.211, but it yielded significant effects of the water restriction factor, *F*(1,10) = 59.68, *p* <.01, η_p_^2^ = 0.856, and its interaction with the operant schedule, *F*(1,10) = 9.81, *p* <.01, η_p_^2^ = 0.495. Planned comparisons revealed in both FR8 and PROG operant schedules that mice consumed more 10% sucrose solution when they were water restricted in comparison to baseline (*p* <.01 for both schedules).

For the choice group (Fig. 6C–E), the two-way factorial ANOVA only revealed a significant effect of water restriction, *F*(1,10) = 18.19, *p* <.01, η_p_^2^ = 0.645, but the effect of operant schedule, *F*(1,10) = 0.09, *p* = 0.77, η_p_^2^ = 0.009, and the interaction, *F*(1,10) = 0.64, *p* =.439, η_p_^2^ = 0.061, were not significant. For the 10% sucrose intake variable the results were basically the same; the two-way factorial ANOVA yielded a significant effect of water restriction, *F*(1,10) = 22.78, *p* <.01, η_p_^2^ = 0.695, and a significant effect of operant schedule, *F*(1,10) = 4.84, *p* =.05, η_p_^2^ = 0.327, but no significant interaction between both variables, *F*(1,10) = 0.574, *p* =.46, η_p_^2^ = 0.054. Finally, for the variable 3% sucrose intake, the two-way factorial ANOVA yielded a significant effect of water restriction, *F*(1,10) = 14.66, *p* <.01, η_p_^2^ = 0.595, but not of operant schedule, *F*(1,10) = 0.89, *p* =.37, η_p_^2^ = 0.082, and no interaction, *F*(1,10) = 1.49, *p* =.25, η_p_^2^ = 0.130. Mice under choice conditions demonstrate that increasing the homeostatic need for water can increase work output, independently of the schedule, and lead to an increase in intake of the free sucrose solution. Thus, water restriction increased all the fluids consumed and the work needed to increase fluid access under both FR8 and PROG choice conditions.

### Phase 5. Analysis of individual differences under “no-choice” and “choice” conditions: Performance based on individual differences in PROG

Average baseline lever-pressing performance in the last week under the PROG operant schedule was analyzed and a median split was used to separate high and low performers (median for the no-choice group = 91.3, and median for the choice group = 82.4). A series of two-way ANOVAs (Operant Schedule [within subjects] × Type of Performer [between groups]) were used to analyze all the dependent variables in each group.

For the “no-choice” experimental condition (Fig. 7A–B), the two-way ANOVA for lever presses showed significant effects of PROG performance level, F(1,9) = 19.50, p <.01, ηp^2^ = 0.684, operant schedule, *F*(1,9) = 19.052, *p* <.01, η_p_^2^ = 0.679, and also there was a significant interaction, *F*(1,9) = 8.062, *p* <.05, η_p_^2^ = 0.473. Planned comparisons showed, as expected, how high performers produced more lever presses compared to the low performers under a PROG operant schedule (*p* <.01). Moreover, high performers pressed the lever more when they were under a PROG operant schedule in comparison to when they were in the FR8 operant schedule (*p* <.01), but this was not the case for the low performers. As for the 10% sucrose intake, the two-way ANOVA revealed a significant effect of PROG performance level, *F*(1,9) = 17.14, *p* <.01, η_p_^2^ = 0.656. Although it approached significance, the operant schedule factor did not reach significance, *F*(1,9) = 4.240, *p* =.07, η_p_^2^ = 0.320, and there was no significant interaction, *F*(1,9) = 0.497, *p* =.498, η_p_^2^ = 0.052. Thus, in general, high performers got access and consumed more of the 10% sucrose independently of the schedule.

The same statistical parameters were used for the “choice” group (Fig. 7C–E). The two-way ANOVA for lever presses did not show significant effects of the operant schedule, *F*(1,9) = 0.01, *p* =.904, η_p_^2^ = 0.002, but it did show a significant effect of the PROG performance level, *F*(1,9) = 10.23, *p* <.05, η_p_^2^ = 0.532, and a significant interaction between both factors, *F*(1,9) = 6.537, *p* <.05, η_p_^2^ = 0.421. Planned comparisons revealed the same pattern that in the “no-choice group”: high performers increased lever presses when the operant schedule involved more effort to obtain the high-reward option (*p* <.05), and they produced more lever presses in the PROG schedule in comparison with low performers (*p* <.05). However, for the 10% sucrose intake, the two-way ANOVA did not show an effect of type of performer, *F*(1,9) = 2.999, *p* =.117, η_p_^2^ = 0.250, or operant schedule, *F*(1,9) = 2.457, *p* = 0.151, η_p_^2^ = 0.214, and no interaction either, *F*(1,9) = 0.577, *p* =.466, η_p_^2^ = 0.060. Finally, for the variable 3% sucrose intake, the two-way ANOVA did not show significant effects: type of performer, *F*(1,9) = 0.007, *p* =.930, η_p_^2^ = 0.00008, operant schedule requirement, *F*(1,9) = 0.008, *p* =.928, η_p_^2^ = 0.00009, or interaction, *F*(1,9) = 0.233,* p* =.640, η_p_^2^ = 0.025.

Thus, in summary, the split between high and low performers based on the last week of PROG performance yielded the same pattern of results across the two experimental conditions (choice vs. no choice) in terms of work output, but not in terms of consumption.

### Analysis of total fluid intake based on individual differences in PROG

Further analysis of the total amount of fluid consumed (Fig. 8) with a three-way factorial ANOVA (Operant Schedule × Type of Performer × Choice Condition) yielded significant differences between choice groups, *F*(1,36) = 22.93, *p* <.01, η_p_^2^ = 0.389, type of performer, *F*(1,36) = 20.23, *p* <.01, η_p_^2^ = 0.360, and Schedule × Choice Condition interaction, *F*(1,36) = 5.01, *p* <.05, η_p_^2^ = 0.122. However, there was no significant effect on the following: operant schedule, *F*(1,36) = 0.09, *p* =.760, η_p_^2^ = 0.003, Operant Schedule × Type of Performer, *F*(1,36) = 0.07, *p* =.78, η_p_^2^ = 0.002, and Choice Condition × Type of Performer, *F*(1,36) = 3.94, *p* =.054, η_p_^2^ = 0.099. There was no three-way interaction, *F*(1,36) = 2.05, *p* =.160, η_p_^2^ = 0.054 (Fig. 8). Thus, in general the “choice” group consumed more total fluid and the high performers consumed more than the low performers independently of the operant schedule. Further analyses were done separating the choice conditions. A two-way factorial ANOVA (Operant Schedule [within subjects] × Type of Performer [between groups]) for the “no-choice” group yielded significant differences for type of performer, *F*(1,9) = 39.90, *p* <.01, η_p_^2^ = 0.816, but there was no significant effect of the operant schedule, *F*(1,9) = 4.47, *p* =.06, η_p_^2^ = 0.332, and no interaction between both factors, *F*(1,9) = 0.92, *p* =.36, η_p_^2^ = 0.093. However, among the “choice” group, a two-way factorial ANOVA did not show significant effects of type of performer, *F*(1,9) = 1.42, *p* =.26, η_p_^2^ = 0.136, nor of operant schedule, *F*(1,9) = 3.58, *p* =.09, η_p_^2^ = 0.285, and no interaction between Operant Schedule × Type of Performer, *F*(1,9) = 2.80, *p* =.13, η_p_^2^ = 0.237. Thus, it seems that when animals can regulate fluid intake by drinking from a freely available solution the difference in willingness to work between high and low PROG performers disappears.

### Body weight progression across weeks

Body-weight data across weeks were analyzed using a two-way factorial ANOVA (Week × Choice Group) starting the last week of FR8 in which both groups had the same experimental conditions (Fig. 9). Results revealed a significant effect of choice condition, *F*(1,21) = 4.64, *p* <.05, η_p_^2^ = 0.181, and a significant effect of week, *F*(11,231) = 5.42, *p* <.01 η_p_^2^ = 0.209, but no interaction, *F*(11,231) = 1.43, *p* =.18, η_p_^2^ = 0.074. These results suggest that intake of the freely available sucrose solution allowed for a bigger increase in body weight, although both groups increased weight across weeks.

## Discussion

The current studies aimed to evaluate, in a new operant paradigm adapted for mice, the impact of several conditions that affect responding and choice when there is a high exertion of effort required for obtaining access to sucrose reinforcement. In the present work, assessment of experimental conditions that introduce effort-based choices was done both between groups (choice of reinforcers vs. no choice), and within groups by changing from FR8 (constant and predictable demand of effort) to a PROG schedule that increases effort demands during the operant session. Another difference between the two schedules is the “time out” in the PROG schedule (if the animal stops lever pressing for 2 min, the session ends) which forces the mice to be more focused on responding in order not to lose access to the preferred reinforcer. An important conclusion of the present results is that the presence of a low effort/free alternative separates the performance of the two groups (choice vs. no choice) as the work requirement is progressively increasing across the session, a difference that was not seen when the work is high but constant (as in the FR8; see Fig. 4A). Moreover, when the homeostatic demands for fluid increase, the performance of both groups of animals increases with a similar pattern as when they had water ad libitum; there was no difference between schedules in the choice group, and a high increase after PROG in the no-choice group (see Figs. 6A and 6 C). This contrast between groups indicates that progressive ratios are the work schedule that have a more profound impact on effort evaluation and spontaneous willingness to work.

In addition, finding those conditions that allow the expression of individual differences in willingness to work is relevant for future studies attempting to find the neural substrate of those differences, and to work on preventive personalized strategies to reduce vulnerability to symptoms like anergia and fatigue. Our results in mice corroborate the effectiveness of progressive ratios for differentiating between the performance of individual animals (Figs. 7A and 7 C). Only the high performers showed an increase in performance from FR8 to PROG in both choice groups. In previous experiments in rats, it has been demonstrated that the use of operant schedules with high effort requirements such as progressive ratios introduces a high degree of variability in the response of different animals, therefore, those schedules are useful for the study of individual differences (Randall et al., 2012, 2014; SanMiguel et al., 2018). In addition, the use of sucrose solutions as the natural reinforcer in animals that are normally not restricted of water also adds an element in the decision-making process that emphasizes the evaluation of the effort required as the most important factor, since the homeostatic value of the reinforcer may not be very strong. Thus, individual variability in willingness to exert effort can be expressed more spontaneously.

In terms of the consumption patterns, when comparing experimental conditions (choice vs. no-choice groups), the pattern of results shows that in the more traditional operant paradigm (the no-choice group), animals show increased work output when PROG is introduced (Fig. 4A), and their performance enables them to maintain the amount of the sucrose solution at the same level that they had during the less demanding task (FR8). Moreover, in the no-choice group there was a significant positive correlation between 10% sucrose consumption on both schedules (Fig. 5A). However, these animals in general consume less total fluid compared to the choice group (Fig. 8), which could be related to a more blunted body weight gain across weeks (see Fig. 9). Thus, when water restriction increases the homeostatic value, work output during PROG increases (much less so during FR8), so no-choice mice are able to achieve a higher level of fluid consumption than under no restriction conditions. The fluid consumed under PROG-restriction is no different from FR8-restriction, thus showing the same adjustment than occurred under the no restriction conditions.

The availability of a low effort option for obtaining reinforcement (in the choice group) changed the overall pattern of responding. The presence of the free 3% concentration was able to significantly reduce lever pressing reinforced by the 10% sucrose solution (Fig. 3A). Animals consumed around 0.7 ml of 10% sucrose provided by lever pressing and around 0.7 ml of the free 3% concentration, see third bar in Fig. 3B and second bar in Fig. 3C). However, there was a much higher consumption of the 10% solution when both concentrations were freely available, indicating a strong preference for this concentration when there was no effort requirement in place (Fig. 3D), and a higher capacity for volume consumption (total of 1.8 ml, approximately). Other authors also have used concentrations of sucrose between 2 and 5% as a low value alternative (Bailey et al., 2020; Fry et al., 2021; Robles & Johnson, 2017). The baseline rate of lever pressing in the choice group is much lower, especially in the highly demanding task (PROG); choice animals reached a much lower ratio than animals in the no-choice group (25 vs. 70; Fig. 4C), and the sucrose consumption that depends on operant performance (10%), showed no significant correlation across the two schedules (Fig. 5A). This big difference in the higher ratio achieved between the no-choice and the choice conditions has been previously observed with similar procedures in rats using food as the reinforcer, in which animals trained in a progressive lever pressing procedure, when changed to a progressive ratio-choice situation, reduced significantly their maximum ratio achieved (Schweimer & Hauber, 2005).

When water is restricted, the choice group increases lever pressing but this change is minimal since they compensate by increasing also free sucrose consumption (Fig. 6E). The present results involving water restriction are consistent with results from satiation manipulations in mice and rats (Pardo et al., 2015; Randall et al., 2012; Yang et al., 2020a, 2020b, 2020c). In the present results, when reducing the amount of water available in the home cage, we observed an increase in work output (in both choice conditions) consistent with an increase in the homeostatic value of the reinforcer (a fluid), and of course, also an increase in the free fluid intake. Consistent with the present results, mice tested on a touch-screen task, and preexposed ad libitum to food (reduction of the homeostatic value), reduced not only panel presses (the high-effort option) but also the consumption of the free pellets that were concurrently available (Yang et al., 2020a, 2020b, 2020c).

When analyzing the impact of these experimental condition on individual differences in terms of willingness to work, we found similar patterns in both settings, but more information from the choice group. Significant increments in lever pressing from FR8 to PROG schedules emerge in both experimental conditions (in the choice condition, there were no differences when all the animals were analyzed together; Fig. 4A). However, as shown by the individual data points tracking the change from FR8 to PROG, these differences were only seen among the high responders in any of the choice conditions. The low performers either stayed the same (the no-choice condition; Fig. 7A) or even showed a decrease in lever pressing when presented with a much more demanding schedule (Fig. 7C). The no-choice condition is marked by a big difference between performers based on PROG performance, and a clear parallel between lever pressing and amount of reinforcer consumed. Thus, willingness to work parallels efficiency in sucrose consumption accessible after the ratio is completed in this group. However, high PROG performers among the choice group did not show significant differences in sucrose consumed compared to low performers (Figs. 7D–E), probably because both types of performers have learned to compensate for their intake with the free option, showing the potential impact of previous experience on the behavioral pattern of responding. Thus, the choice condition allows the manifestation of individual differences in performance, but no difference in total volume consumed (Fig. 8), indicating that at least in mice the choice condition induces individual differences based mostly on willingness to work, rather than the inclination to obtain the maximum amount of reinforcer. Future research should confirm this point by using experimental settings in which the instrumental response has intrinsic reinforcing properties based on behavioral activation such as running in a running wheel.

While studies of effort-based decision making were originally established in rats, there is an emerging literature on the use of operant versions of effort-related tasks in mice (Heath et al., 2015, 2016; López-Cruz et al., 2021; Phillips et al., 2018; Robles & Johnson, 2017; Yang et al., 2020a, 2020b, 2020c). The application of rigorous and appropriate behavioral animal models is important for the study of the neurobiological bases of normal behavioral phenotypes, but it is also relevant because it can reveal phenotypes with specific vulnerabilities, and can serve as the tools for establishing therapeutic targets. The lever pressing effort-based choice task described in the present work offers a useful procedure for assessing this important aspect of motivational function in mice, which could have translational implications for studying motivational dysfunctions in humans such as avolition in schizophrenia or fatigue and anergia in depression (Markou et al., 2013; Salamone & Correa, 2002, 2012).

## Supplementary Information

Below is the link to the electronic supplementary material.

Supplementary file1 (DOCX 985 kb)

## Data Availability

The data for the experiments would be made publicly available at the institutional repository upon acceptance of the manuscript. None of the experiments was preregistered.
