# Supplementary material for: Willingness to work for sucrose: Impact of schedules, reinforcer alternatives, homeostatic value, and individual differences in male mice
Source: Learn Behav. 2025 Nov 6;54(2):229–42. doi: 10.3758/s13420-025-00695-y (PMC13194295; doi:10.3758/s13420-025-00695-y)
Supplement: Supplementary file 1 — Supplementary file1 (DOCX 985 kb) [file 13420_2025_695_MOESM1_ESM.docx]

SUPPLEMENTAL MATERIAL

**
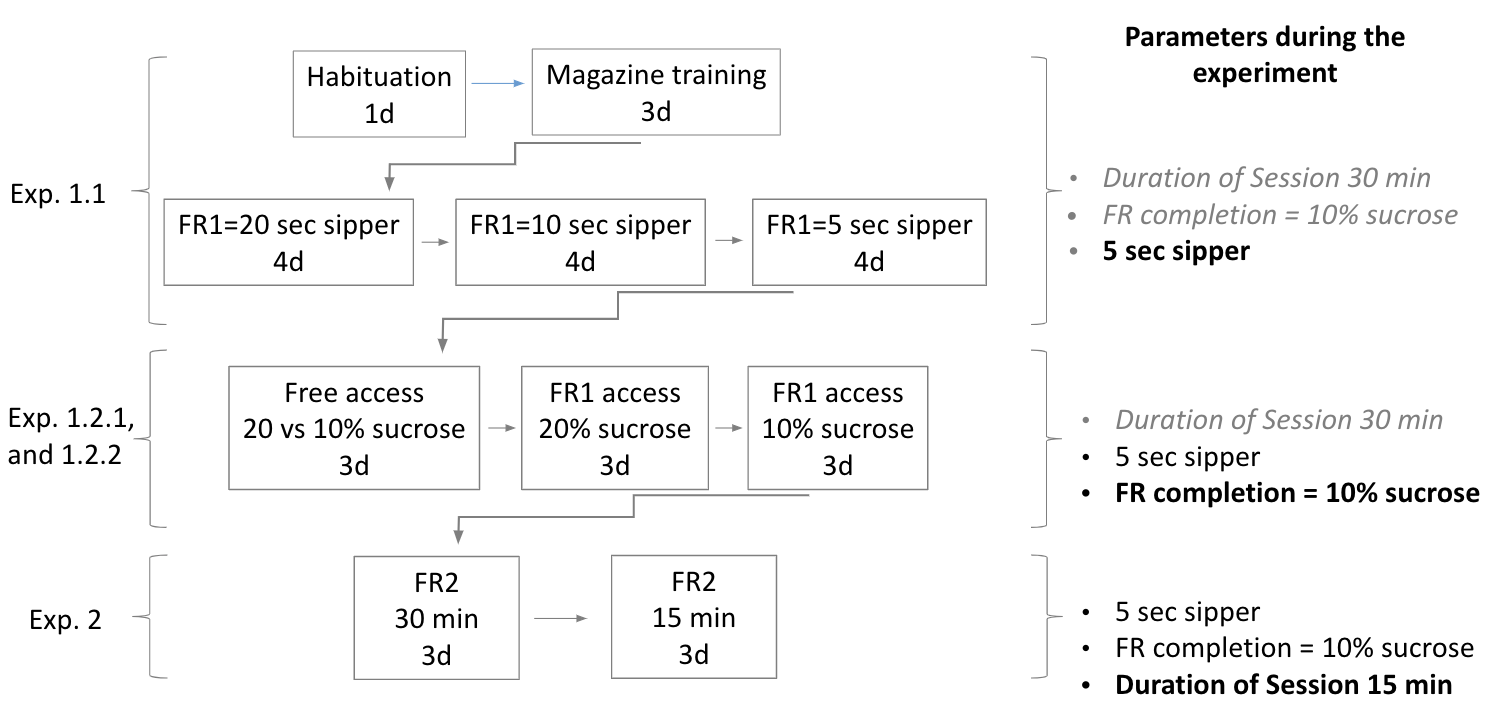
**

**Supp. fig.1.** Schematic of the experimental parameters being tested, and time sequence.


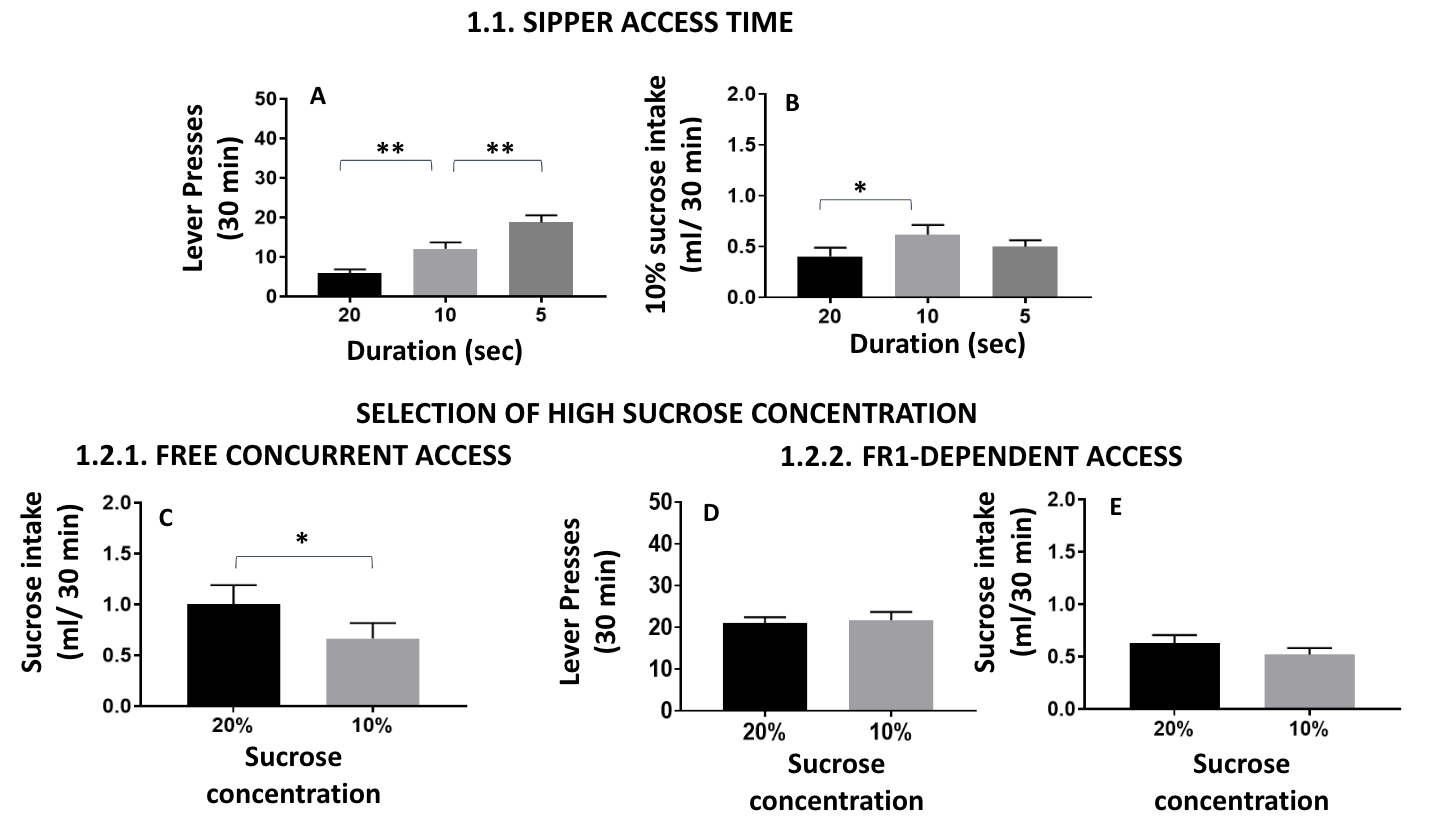


**Supp. fig. 2.** Upper part: Effect of access time to the reinforcer on lever presses (A) and 10% sucrose intake (B). Lower part: Effect of sucrose concentration under free concurrent presentation on sucrose intake (C), or under FR1 responding: lever presses (D), and sucrose intake (E). Bars represent the mean ± SEM number of lever presses or ml consumed during 30 minutes. *p<0.05, **p<0.01 significant differences between groups.

**
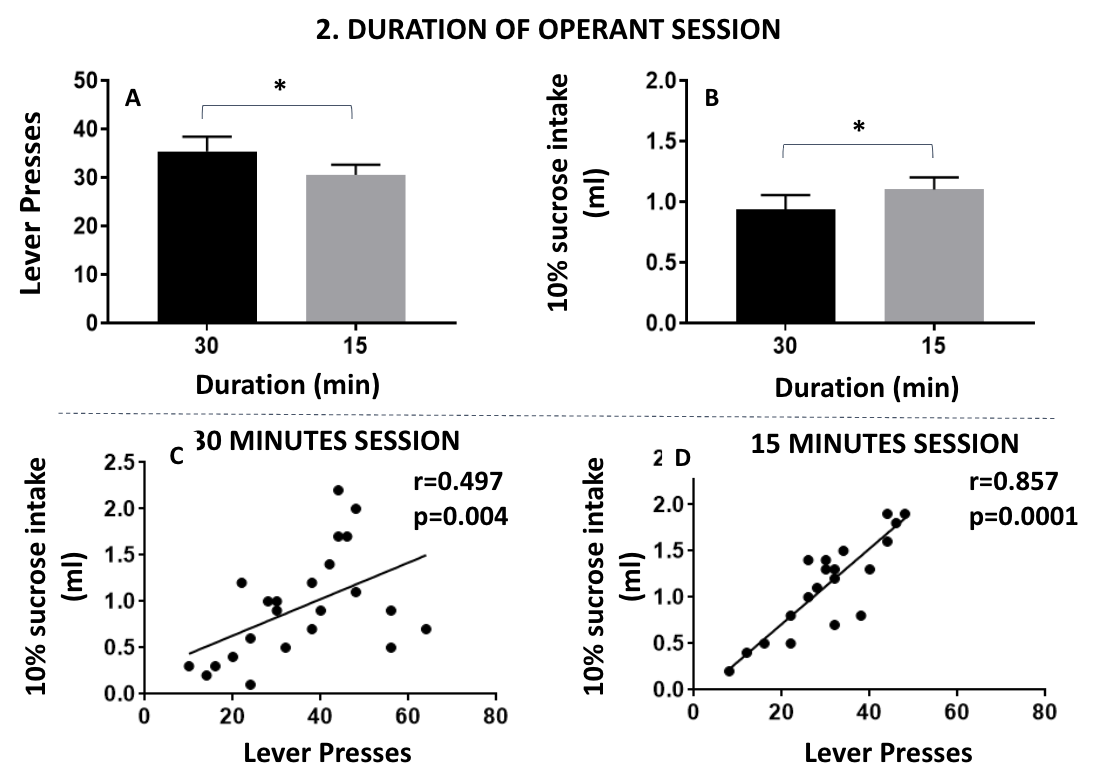
**

**Supp. fig. 3.** Upper part: Effect of introducing free choice conditions on FR8 lever presses (A), operant-dependent 10% sucrose intake (B) and free sucrose intake (C). Lower part: Effect of concurrent free access presentation in the operant box of two concentrations of sucrose (10 and 3%) on volume consumed in a session of 15 minutes. Bars represent the mean ± SEM of lever presses or ml consumed. *p<0.05, **p<0.01, significant differences between experimental conditions.


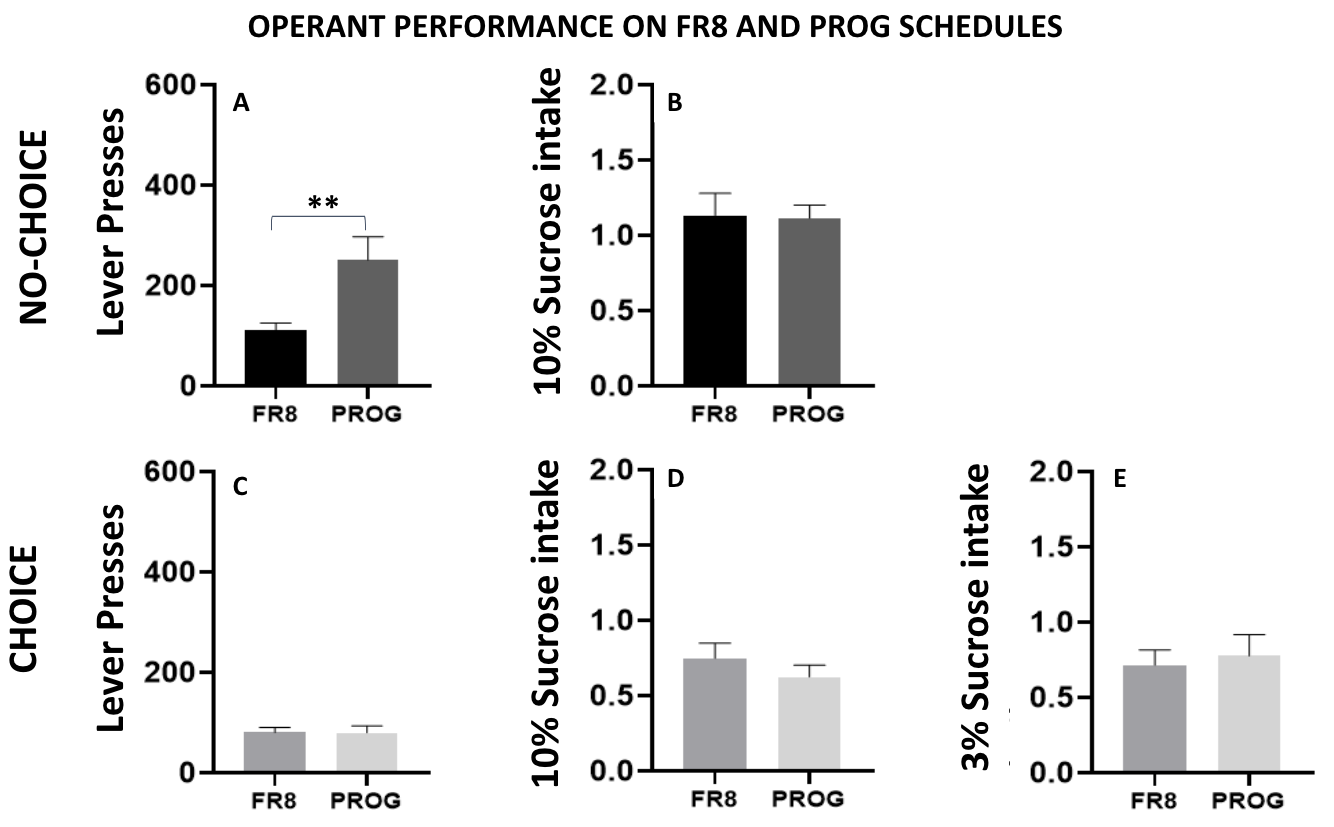


**Supp. fig. 4.** Average performance during 5 weeks of FR8 followed by 5 weeks of PROG schedules. Bars represent the mean ± SEM total lever presses (A, C), ml of 10% sucrose consumed (B, D), and ml of 3% sucrose consumed (E) in 15 minutes sessions. **p<0.01, significant differences between operant schedules.


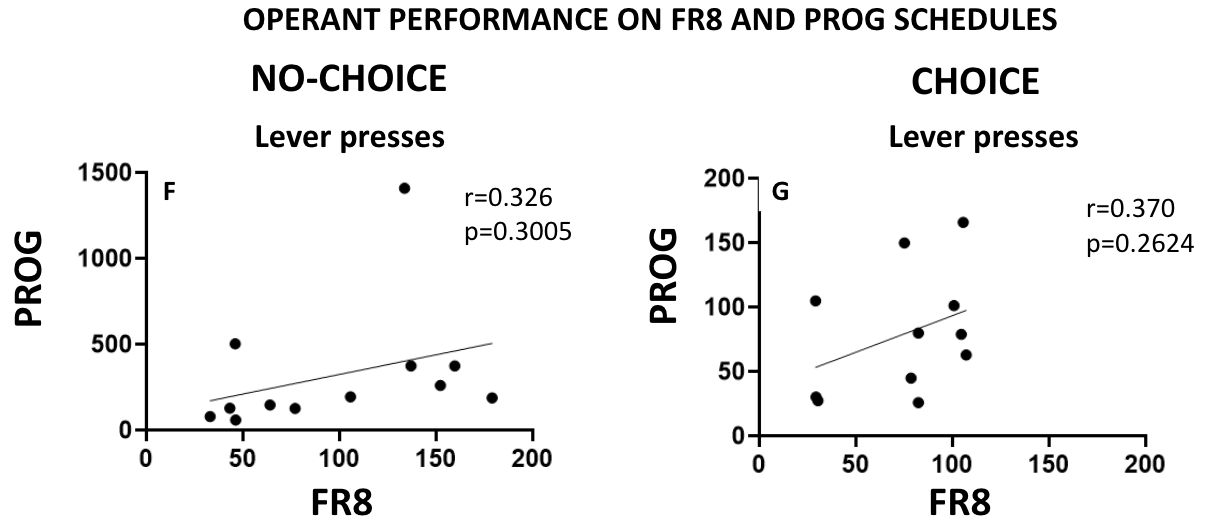


**Supp. fig. 5.** Correlation between the average of the lever presses for every mice during the last week of each schedule. Individual data for the no-choice group (A), and the choice group (B). in 15 minute sessions.


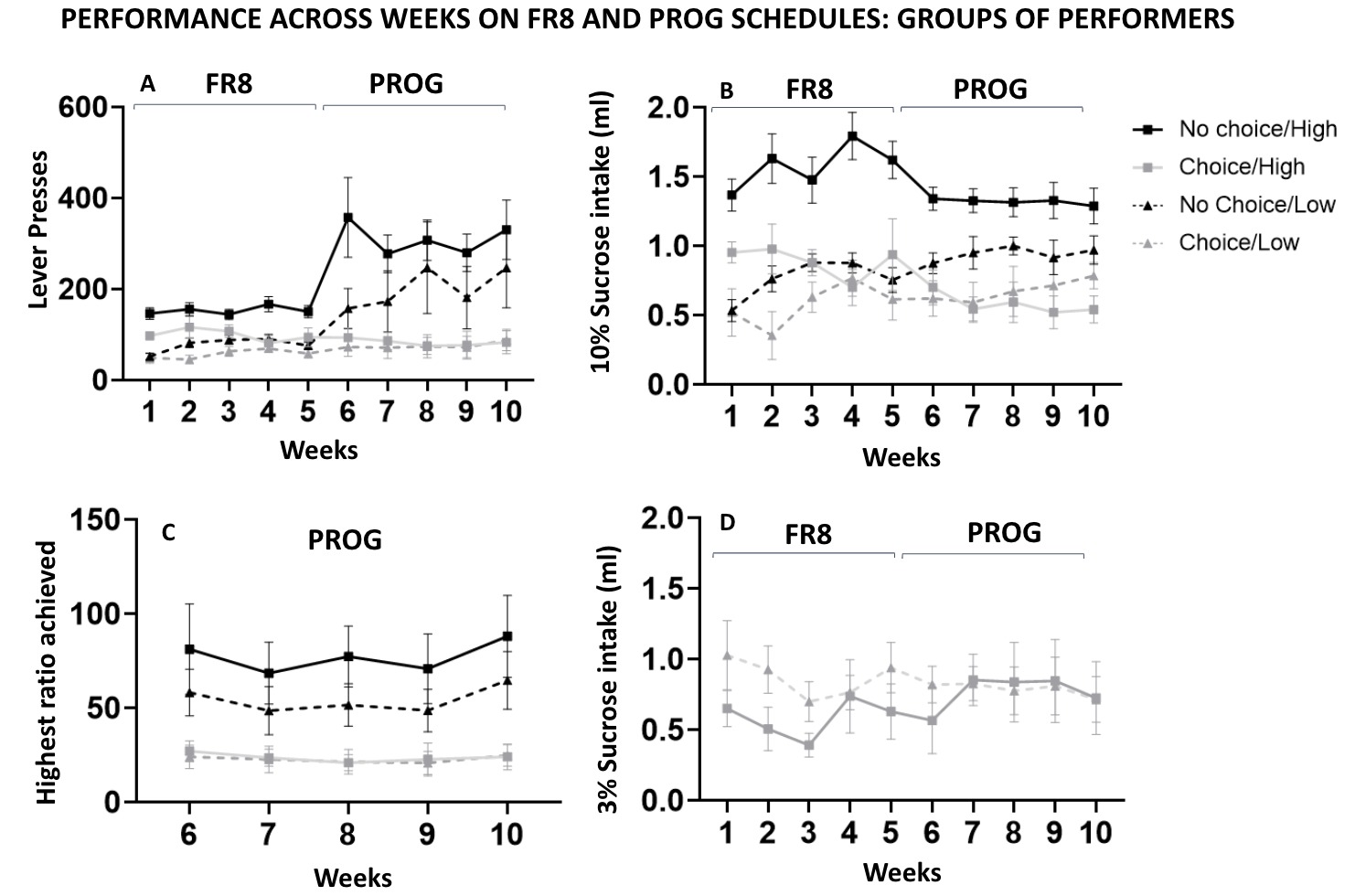


**Supp. fig. 6.** Effect of choice conditions during operant sessions with different work requirements (FR8 and PROG schedules), according to type of performer (N=22). Data represent the mean ± SEM number of lever presses (A), 10% sucrose ml consumed (B), highest ratio achieved during the PROG weeks (C), and free 3% sucrose ml consumed for the choice groups (D) in 15 minutes sessions.

For these results, a three-way repeated measures ANOVA was conducted to evaluate the effects of Week (within-subject factor), Choice condition ("Choice" vs "No Choice"), and Type of Performer ("High Performer" vs "Low Performer") on Lever Presses, 10% sucrose intake, and highest ratio achieved.

For Lever Presses, there was a significant main effect of Week (F(9, 162)=6.650, p<0.001, η²p=0.270), a significant main effect of Choice condition (F(1, 18) = 17.801, p<0.001, η²p =0.497) and of Type of Performer (F(1, 18)=5.351, p=0.033, η²p =0.229). There was also a significant interaction between Week and Choice condition (F(9, 162)=6.317, p<0.001, η²p=0.260), while the interactions between Week and Type of Performer (F(9, 162)=0.548, p=0.837, η²p=0.030), between Choice condition and Type of performer was not significant (F(1, 18)=1.784, p=0.198, η²p=0.090), and the three-way interaction (F(9, 162)=0.698, p=0.710, η²p=0.037) were not significant.

For 10% sucrose intake, there was a significant main effect of Choice condition (F(1, 18)=24.250, p<0.001, η²p=0.574) and Type of Performer (F(1, 18)=13.657, p=0.002, η²p=0.431), but no main effect of Week (F(9, 162)=1.773, p=0.077, η²p=0.090). There was a significant interaction between Week and Type of Performer (F(9, 162)=7.215, p<0.001, η²p=0.286), as well as a significant interaction between Choice condition and Type of Performer (F(1, 18)=6.582, p=0.019, η²p=0.268), while the interaction between Week and Choice condition (F(9, 162)=1.387, p=0.198, η²p=0.072), and the three-way interaction (F(9, 162)=1.225, p=0.283, η²p=0.064) were not significant.

For the highest ratio achieved, there was a significant main effect of Choice condition (F(1, 18)=12.663, p=0.002, η²p=0.413), but the main effect of Type of Performer (F(1, 18)=0.984, p=0.334, η²p=0.052) and Week factor (F(4, 72)=2.211, p=0.076, η²p=0.109) were not significant. The two way-interaction between Choice condition and Type of Performer (F(1, 18)=0.842, p=0.371, η²p=0.045), Week and Choice condition (F(4, 72)=1.209, p=0.315, η²p=0.063), or Week and Type of Performer (F(4, 72)=0.032, p=0.998, η²p=0.002), as well as the three-way interaction (F(4, 72)=0.071, p=0.991, η²p=0.004) were not significant.

Finally, a two-way repeated measures ANOVA was conducted for 3% sucrose intake, showing no significant main effect of Week (F(9, 81)=0.870, p=0.556, η²p=0.088), Type of Performer (F(1, 9)=0.524, p=0.487, η²p=0.055), and no interaction between Week and Type of Performer (F(9, 81)=1.000, p=0.447, η²p=0.100).


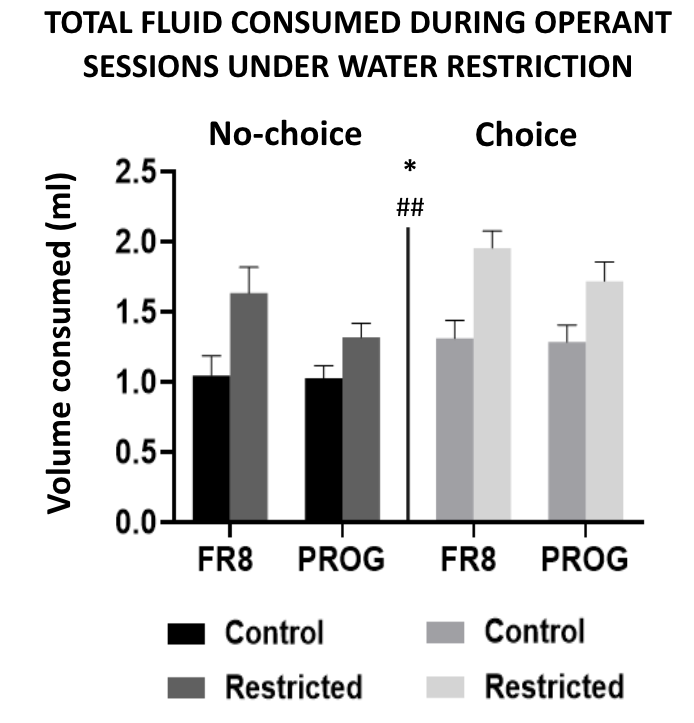


**Supp. fig. 7.** Total volume consumed during the operant session comparing both choice groups. ##p<0.01 denotes significance for the main factor water restriction. *p<0.05 denotes significance for the main factor operant schedule in a three way ANOVA: type of restriction x operant schedule x choice condition.
